# MPC-ESO Position Control Strategy for a Miniature Double-Cylinder Actuator Considering Hose Effects

**DOI:** 10.3390/mi14061201

**Published:** 2023-06-06

**Authors:** Tengfei Ma, Bin Wang, Zhenhao Wang

**Affiliations:** 1 College of Energy and Power Engineering, Nanjing University of Aeronautics and Astronautics, Nanjing 210016, Chinawzh0317@nuaa.edu.cn (Z.W.); 2 Jiangsu Province Key Laboratory of Aerospace Power System, Nanjing University of Aeronautics and Astronautics, Nanjing 210016, China

**Keywords:** position control, miniature hydraulic actuator, model predictive control, extended state observer, generalized regression neural network

## Abstract

Miniature hydraulic actuators are especially suitable for narrow-space and harsh environment arrangement. However, when using thin and long hoses to connect components, the volume expansion caused by pressurized oil inside can have significant adverse effects on the performance of the miniature system. Moreover, the volumetric variation relates to many uncertain factors that are difficult to describe quantitatively. This paper conducted an experiment to test the hose deformation characteristics and presents the Generalized Regression Neural Network (GRNN) to describe the hose behavior. On this basis, a system model of a miniature double-cylinder hydraulic actuation system was established. To decrease the impact of nonlinearity and uncertainty on the system, this paper proposes a Model Predictive Control (MPC) based on Augmented Minimal State-Space (AMSS) model and Extended State Observer (ESO). The extended state space acts as the prediction module model for the MPC, and the disturbance of the ESO estimates is fed to the controller to improve the anti-disturbance capability. The full system model is validated by comparison between the experiment and the simulation. For a miniature double-cylinder hydraulic actuation system, the proposed MPC-ESO control strategy contributes to a better dynamic than conventional MPC and fuzzy-PID. In addition, the position response time can be reduced by 0.5 s and achieves a 4.2% reduction in steady-state error, especially for high-frequency motion. Moreover, the actuation system with MPC-ESO exhibits better performance in suppressing the influence of the load disturbance.

## 1. Introduction

Due to the outstanding advantages in power-weight ratio and response capacity, hydraulic actuators have been widely used in many fields [1,2]. Small-scale hydraulic actuators are suitable for an extremely narrow-space arrangement, such as aircrafts, wind tunnels, or robotics [3]. For this kind of hydraulic system, uncertainty can bring about adverse effects on the system control performances [4,5]. In these hydraulic systems, hoses play a crucial role as they connect adjacent hydraulic components and facilitate the transmission of pressurized fluid to generate power. However, hoses in hydraulic systems introduce uncertainties due to several key factors [6]. The nonlinearity of hose behavior, characterized by the nonlinear relationship between input pressure and volumetric expansion, results in variations and uncertainties in system response. Material properties, including elasticity, deformation characteristics, and aging, contribute to further uncertainties in hose behavior. The uncertainties present a great challenge to the component model and control strategy of the hydraulic system requiring capabilities of fast response, high precision, and strong anti-disturbance [7,8]. Accordingly, reliable hose modeling and excellent control strategy for small-scale hydraulic actuation systems are particularly important.

Some studies focus on hydraulic hose models. Lumped Parameter Method (LPM) is a typical case [9]. Due to the coupling of nonlinearity and uncertainty with other components, LPM cannot characterize the time-varying pressurized chamber of a hose across all conditions. Therefore, there is a pressing need for more effective hose modeling technology, especially for the systems with model-based control strategies. Researchers have conducted many studies on the application of Machine Learning (ML) in material modeling, including the search for high-performance Ferroelectric Tunnel Junctions (FTJs) and the prediction of dielectric strength in LDPE/MgO materials. ML techniques, such as the gradient boosting classification model and Artificial Neural Networks (ANNs), demonstrate their effectiveness in guiding FTJ exploration and accurately predicting material properties [10,11,12]. These findings highlight the wide application of ML to material modeling and property prediction in different fields. As a radial basis function network based on nonlinear regression analysis, GRNN has strong nonlinear mapping capacity and learning speed than conventional Radial Basis Neural Network (RBNN) [13,14]. These characteristics make GRNN particularly well-suited for accurately representing the time-varying behavior of the pressurized chamber within hoses across various operating conditions. Therefore, GRNN is a reliable and effective approach to modeling hoses.

In terms of position control of hydraulic actuation systems, PID has been commonly used due to its simple structure and easy implementation [15]. On this basis, fuzzy PID was developed for better steady-state accuracy and dynamic response [16]. Aiming at the time-varying, low-damping, nonlinear characteristics and external disturbance in hydraulic systems, fuzzy PID optimizes the controller parameters online for improving the dynamic and static characteristics [17].

In the early 1980s, the concept of MPC algorithm was first proposed as an online optimized algorithm to solve nonlinear problems with constraints and time delays. At present, MPC has been employed in some fields, such as vehicles and motors [18,19]. Despite better tracking, the expectation of MPC highly depends on the model. Considering the adverse effect of the hose deformation on the system, it is difficult for the existing MPC strategy to obtain satisfactory control performance, especially under the action of external disturbances. In order to improve the prediction results and enhance the system performance, some MPC-based algorithms were explored in recent years. Some researchers have developed new control strategies for Electromagnetic Actuators (EAs) to outperform mechanical actuation. MD Shakib Hasan proposed a novel Model Predictive Control (MPC) algorithm that combines a Finite Set-MPC (FS-MPC) controller, a Proportional Integral (PI) controller, and a Kalman estimator-based state estimator. Simulation results on an EA model demonstrate improved performance compared to traditional approaches [20]. In another investigation, researchers proposed a servo motor-driven hydraulic system for injection molding machines. They employed an MPC approach based on neurodynamic optimization, achieving satisfactory performance and faster optimization. This method holds promise for fast response process applications [21]. Combining an Extended State Observer (ESO) with Model Predictive Control (MPC) offers a promising approach to overcome the limitations of MPC and improve control performance. The ESO estimates real-time disturbances and compensates for system uncertainties, while MPC optimizes control actions based on a predictive model [22]. This integration enhances robustness, disturbance rejection, and tracking performance, making it a valuable strategy for control system improvement. 

In this paper, we focus on the characterization of volumetric variation in a hose accommodating pressurized fluid in a hydraulic system. The proposed approach utilizes the GRNN method for hose modeling and combines MPC with an ESO strategy for position tracking control in a miniature double-cylinder hydraulic actuation system. The GRNN provides a nonlinear mapping capacity and fast learning speed, enabling efficient predictive control for complex plants. The MPC-ESO controller compensates for uncertainties and external disturbances, enhancing system performance. The effectiveness of the proposed controller is demonstrated through simulations and experiments on an experimental system. The results confirm the reliability of the model and the improved control performance achieved. This research contributes to the development of reliable hose modeling and control strategies for miniature hydraulic actuators, offering potential applications in various fields.

## 2. Arrangement and Model

### 2.1. System Arrangement

**Figure 1:**
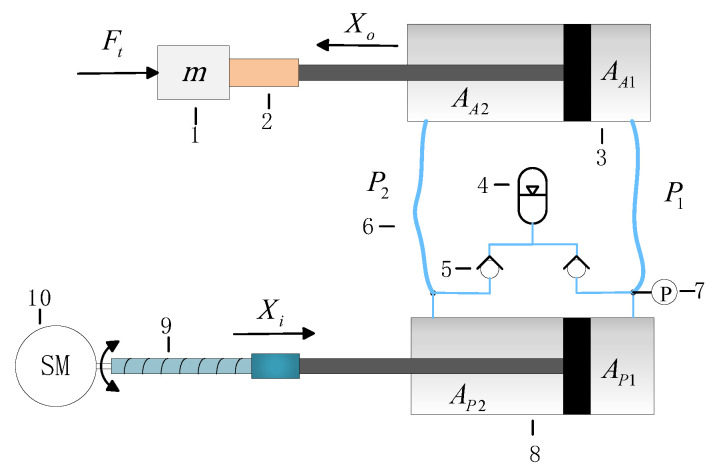
Arrangement diagram of the actuation system. 1. Load mass; 2. LVDT; 3. Actuating cylinder; 4. Accumulator; 5. Check valve; 6. Hose; 7. Pressure sensor; 8. Power cylinder; 9. Ball screw; 10. Servo motor.

Figure 1 shows the arrangement diagram of the double-cylinder actuation system. The electric motor rotates when receiving a pulse input, and a ball screw converts the rotation of the motor into linear motion. When the piston of the power cylinder extends or retracts, it makes the piston of the actuating cylinder perform the same action by pressurized oil in the corresponding chamber. The flexible hoses connect the two cylinders, which allows for narrow-space arrangement and remote control for the actuating cylinder. In addition, an accumulator rather than the conventional oil source design greatly simplifies the system structure and improves the transient performance [23].

### 2.2. System Mathematical Model

**Figure 2:**
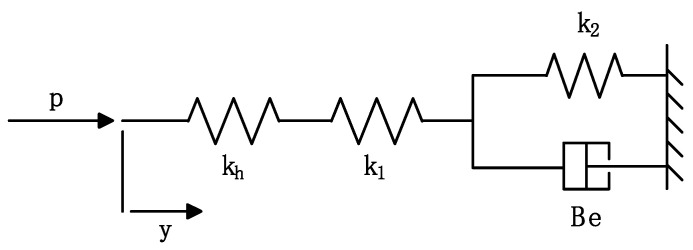
Viscoelastic model for hose.

In order to build the state space model of the double-cylinder actuator system, the hose is mathematically described based on the viscoelastic model (as shown in Figure 2). Generally, the hose has significantly larger expansion than the pipe if filled with high pressure oil. When modeling the hose, both the static and dynamic variation caused by the pressure and the viscoelasticity of the hose material are considered. The viscoelastic behavior of a hose can be represented using a combination of spring and damping elements. The spring model captures the elastic properties of the hose, where the generated force is proportional to the deformation. The damping element represents the viscous properties of the hose, where the generated force is proportional to the deformation velocity. The damping coefficient represents the internal viscous resistance of the hose.

The relationship between Δ*p* and Δ*y* can be described by:

(1) $$\frac{\Delta p}{\Delta y}=k\cdot \frac{T_{1}s+1}{T_{2}s+1}$$

(2) $$\begin{array}{l} \frac{1}{k}=\frac{1}{k_{1}}+\frac{1}{k_{2}}+\frac{1}{k_{h}}\\ k_{h}=2\frac{\beta_{e}}{a}\\ T_{1}=\frac{Be}{k_{2}},T_{2}=\frac{Be}{k_{2}+k_{1}\cdot k_{2}/(k_{1}+k)} \end{array}$$

where *p* is the pressure inside the hose; *y* is the radius variation of the hose; *k_h_* is the equivalent coefficient of the oil compression converted into elastic deformation of the hose, which quantifies the relationship between the compression of the hydraulic oil and the resulting elastic deformation of the hose; *k*_1_ and *k*_2_ are the elastic coefficients of the hose, which determine how the hose responds to external forces or pressure changes; *Be* is the elastic damping coefficient of the hose, indicating the amount of damping or energy dissipation in the hose’s elastic response; *a* is the average radius of the hose; and *β_e_* represents the volumetric modulus of the hydraulic oil [23].

Increment of the hose volume ∆*V* can be written as:

(3) $$\Delta V=(\pi {\left(a+\Delta y\right)}^{2}-\pi a^{2})l=(2\pi a\Delta y+\pi \Delta y^{2})l$$

where *l* is the length of the hose. By ignoring the minimal delta Δ*y*^2^, Equation (3) is simplified as:

(4) $$\Delta V=2\pi a\Delta yl=2\pi al\frac{\Delta P\cdot (T_{2}s+1)}{k\cdot (T_{1}s+1)}$$

The flow rate equations of the system are written as follows:

(5) $$\left\{\begin{array}{l} A_{P1}{\dot{X}}_{i}-A_{A1}{\dot{X}}_{o}=\frac{2\pi al\cdot (T_{2}s+1)}{k\cdot (T_{1}s+1)}{\dot{P}}_{1}\\ A_{A2}{\dot{X}}_{o}-A_{P2}{\dot{X}}_{i}=\frac{2\pi al\cdot (T_{2}s+1)}{k\cdot (T_{1}s+1)}{\dot{P}}_{2} \end{array}\right.$$

Figure 1 shows that *A_P_*_1_ and *A_P_*_2_ are the effective areas of the power cylinder’s two chambers, while *A_A_*_1_ and *A_A_*_2_ are the effective areas of the actuating cylinder’s two chambers. The piston displacement of the power cylinder is *X_i_* and that of the actuating cylinder is *X_o_*. *F_t_* represents the force due to the external load disturbance; *m* is the equivalent mass of all moving parts; and *P*_1_ and *P*_2_ are the pressures in the rodless and rod chambers of the actuating cylinder, respectively [23].

The kinetics equation of the system is written as:

(6) $$m{\ddot{X}}_{o}=A_{A1}P_{1}-A_{A2}P_{2}-F_{t}$$

The time-varying physical constraint is expressed as:

(7) $${\dot{X}}_{o}\leq r_{0}$$

where *r*_0_ is the expected target value of the system.

*V_h_*_1_ and *V_h_*_2_ are the volume of the hose connecting the rodless chambers and the rod chambers of two cylinders, respectively. They can be described by:

(8) $$\left\{\begin{array}{c} {\dot{V}}_{h1}=\frac{2\pi al\cdot (T_{2}s+1)}{k\cdot (T_{1}s+1)}{\dot{P}}_{1}{}_{}\\ {\dot{V}}_{h2}=\frac{2\pi al\cdot (T_{2}s+1)}{k\cdot (T_{1}s+1)}{\dot{P}}_{2} \end{array}\right.$$

In the MPC control structure, the predictive model is essential for anticipating the system’s future behavior and optimizing control actions. It enables the controller to simulate future trajectories, optimize control sequences, handle uncertainties, and make informed decisions to the achieve desired control objectives. Therefore, based on Equation (5) to Equation (8), the mathematical model of the actuation system is established as:

(9) $$\left\{\begin{array}{l} m{\ddot{X}}_{o}=A_{A1}P_{1}-A_{A2}P_{2}-F_{t}\\ A_{P1}{\dot{X}}_{i}-A_{A1}{\dot{X}}_{o}=\frac{2\pi al\cdot (T_{2}s+1)}{k\cdot (T_{1}s+1)}{\dot{P}}_{1}\\ A_{A2}{\dot{X}}_{o}-A_{P2}{\dot{X}}_{i}=\frac{2\pi al\cdot (T_{2}s+1)}{k\cdot (T_{1}s+1)}{\dot{P}}_{2} \end{array}\right.$$

Let *Y* = *y* = *X_o_* and *U* = *u* = ${\dot{X}}_{i}$, substituting *C* into Equation (9) and the differential equation of the system yields:

(10) $$\dddot{Y}+\frac{1}{T_{2}}\ddot{Y}+\frac{b_{1}T_{1}}{mT_{2}}\dot{Y}+\frac{b_{1}}{mT_{2}}Y=(\frac{b_{2}T_{1}}{mT_{2}}-\frac{F_{t}}{m})\dot{U}+\frac{b_{2}-F_{t}}{mT_{2}}U$$

where,

(11) $$\left\{\begin{array}{c} b_{1}=\frac{(A_{A1}^{2}+A_{A2}^{2})k}{2\pi al}\\ b_{2}=\frac{(A_{A1}^{}A_{P1}+A_{A2}^{}A_{P2})k}{2\pi al} \end{array}\right.$$

Based on the previous equations and assumptions, the control-oriented model can be represented by the following three-order state-space model with constraints:

(12) $$\left\{\begin{array}{c} \dot{X}=A_{c}X+B_{c}U\\ Y=C_{c}X\\ U_{\min}\leq U\leq U_{\max}\\ Y_{\min}\leq Y\leq Y_{\max} \end{array}\right.$$

where,

(13) $$X={\left[x_{1},x_{2},x_{3}\right]}^{T}=[y,\dot{y},\ddot{y}+(\frac{F_{t}}{m}-\frac{b_{2}T_{1}}{mT_{2}})u]$$

The coefficient matrices are:

$$\begin{array}{l} A_{c}=\left[\begin{array}{ccc} 0 & 1 & 0\\ 0 & 0 & 1\\ -\frac{b_{1}}{mT_{2}} & -\frac{b_{1}T_{1}}{mT_{2}} & -\frac{1}{T_{2}} \end{array}\right]\;,\;B_{c}=\left[\begin{array}{c} 0\\ \frac{b_{2}T_{1}}{mT_{2}}-\frac{F_{t}}{m}\\ \frac{b_{2}(T_{2}-T_{1})}{mT_{2}^{2}} \end{array}\right]\;,\;C_{c}=\left[\begin{array}{c} 1\\ 0\\ 0 \end{array}\right]\;,\;D_{c}=0\\ U_{\min}=0,U_{\max}=\infty ,Y_{\min}=0,Y_{\max}=r_{0} \end{array}$$

The developed state-space model will be utilized for the design of the MPC-ESO controller. However, the values of *k*_1_, *k*_2_, and *Be* in the viscoelastic model of the hose are difficult to determine and usually rely on empirical selection. This introduces uncertainty into the behavior model of the double-cylinder actuator, ultimately affecting the performance of the controller. Accordingly, GRNN is proposed to build the hose model, aiming to improve the reliability of the system model.

## 3. Principle and Hose Modeling

### 3.1. GRNN Structure and Principle

Due to high accuracy and fast convergence, GRNN can describe the relationship between the pressure and volumetric variation of the hose cavity.

**Figure 3:**
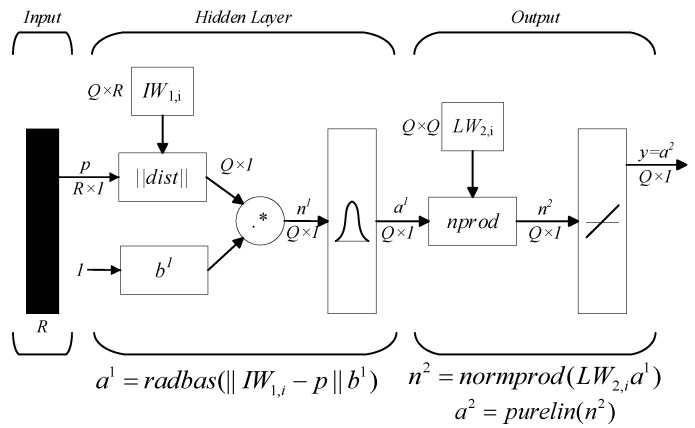
Structure of GRNN.

With a standard three-layer network structure, GRNN is composed of an input layer, a hidden layer, and an output layer, as shown in Figure 3. *P* is the input sample matrix, *R* is the dimension number of *P*, and *Q* is the number of training samples. The Euclidean distance from the input matrix *Q* to the center of the radial basis is used as the input of the hidden layer, and the transfer function of the hidden layer is a Gaussian function with powerful activation characteristics for the network input information [24].

Theoretically, GRNN is a kind of nonlinear regression analysis. *X* is an 𝑛-dimensional random variable (also called a random vector) and *Y* is a random variable. Let *f* (*x*, *y*) be the joint probability density function of variables *X* and *Y* [25]. Assuming that the observed value of variable *X* is *X*, the conditional mean value of *Y* against *X* is:

(14) $$\hat{Y}(X)=E(Y|X)=\frac{\int_{-\infty }^{\infty }yf(X,y)dy}{\int_{-\infty }^{\infty }f(X,y)dy}$$

With the Parzen method in non-parametric estimation, the density function *f* (*x*_0_, *y*) is obtained according to Equation (14) and the sample data are notated as [13]. Therefore, *f* (*x*, *y*) can be written as:

(15) $$\hat{f}(X,Y)=\frac{1}{{\left(2\pi \right)}^{\frac{p+1}{2}}\sigma^{\left(p+1\right)}}\cdot \frac{1}{n}\cdot \sum_{i=1}^{n}e^{-d(X,X_{i})}e^{-d(Y,Y_{i})}$$

(16) $$d(X,X_{i})=\frac{{\left(X-X_{i}\right)}^{T}\left(X-X_{i}\right)}{2\sigma^{2}}$$

(17) $$d(Y,Y_{i})=\frac{{\left(Y-Y_{i}\right)}^{2}}{2\sigma^{2}}$$

where *n* is the sample size; *p* is the dimensionality of *X_i_*; and σ is the width coefficient of the Gaussian function. Integrating Equation (15) into Equation (14) and some transformation yields:

(18) $$\hat{Y}(X)=\frac{\sum_{i=1}^{n}\left(e^{-d(X,X_{i})}\int_{-\infty }^{\infty }ye^{-d(Y,Y_{i})}dy\right)}{\sum_{i=1}^{n}\left(e^{-d(X,X_{i})}\int_{-\infty }^{\infty }e^{-d(Y,Y_{i})}dy\right)}$$

where $\hat{Y}(X)$ is the weighted average of all observations *Y_i_*. Due to $\int_{-\infty }^{\infty }te^{-t^{2}}dt=0$, when two integrations are executed therein, Equation (18) can be simplified as:

(19) $$\hat{Y}(\hat{X})=\frac{\sum_{i=1}^{n}\left(Y_{i}e^{-d(x_{0},x_{i})}\right)}{\sum_{i=1}^{n}e^{-d(x_{0},x_{i})}}$$

Equation (19) is the basic algorithm for the GRNN.

**Figure 4:**
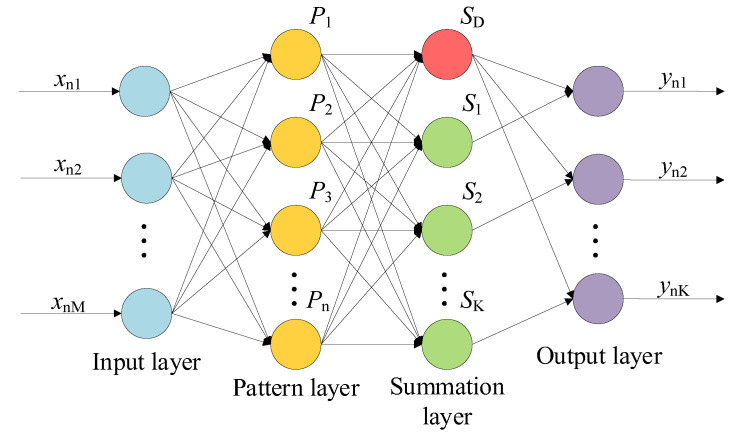
GRNN topology.

Further splitting the hidden layer in Figure 3, GRNN can be divided into four layers: Input layer, pattern layer, summation layer, and output layer [26]. The GRNN structure with *n* number of training samples, *M* training sample input dimension, and 𝐾 output dimension is shown in Figure 4.

The input and output of the network are $X={\left[x_{n1},x_{n2},\cdots ,x_{nM}\right]}^{T}$ and $Y={\left[y_{n1},y_{n2},\cdots ,y_{nK}\right]}^{T}$, respectively.

1. Input layer

The number of neurons in the input layer is equal to the training sample input dimension *M*. Each neuron is a simple distribution unit, directly transmitting the input variables to the pattern layer.

2. Pattern layer

The neuron of the pattern layer is a radial basis function. Transfer function of the neurons can be written as follows:

(20) $$P_{i}=exp[\frac{-{\left(X-X_{i}\right)}^{T}(X-X_{i})}{2\sigma^{2}}]\;i=1,2,\cdots ,n$$

where *X_i_* represents the i*_th_* input sample.

3. Summation layer

The summation layer includes two types of neurons. The denominator in Equation (19) stands for an arithmetical summation of the outputs of all neurons in the pattern layer. The transfer function for this neuron can be written as:

(21) $$S_{D}=\sum_{i=1}^{n}P_{i}$$

The numerator in Equation (19) stands for a weighted summation of the outputs of all neurons in the pattern layer. The connection weight is *y_jk_*, which is the *k_th_* element in the *j_th_* output vector in the training sample, and the transfer function of this neuron can be written as:

(22) $$S_{nk}=\sum_{j=1}^{n}y_{jk}P_{nj}$$

where *k* represents the *k_th_* molecule summation neuron, *k* = 1,2, …, *K*.

4. Output layer

The number of the neurons of the output layer is equal to K, the dimension of the output variables in the learning samples. The final output value of Neuron *j* based on Equation (19) is:

(23) $$y_{j}=\frac{S_{nk}}{S_{D}}$$

### 3.2. Hose Model

**Figure 5:**
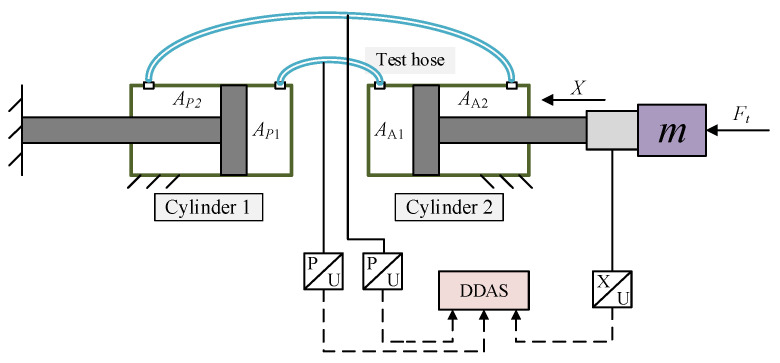
Schematic diagram of the test system.

As no model can precisely characterize the pressurized hose used in Figure 1, the actuation system is remade to collect the deformation data of the hose. Figure 5 shows the schematic of the testing system of the hose characteristics.

It includes two pressure sensors, one displacement sensor, two hydraulic cylinders, and two tested hoses. The pressure sensors measure the pressure in two hoses. The position of the actuating cylinder is collected by the displacement sensor. The hose pressure and the piston displacement are collected as raw data by the Digital Data Acquisition System (DDAS). A loading system exerts a flexible load on the piston of actuating cylinder to move the piston.

**Figure 6:**
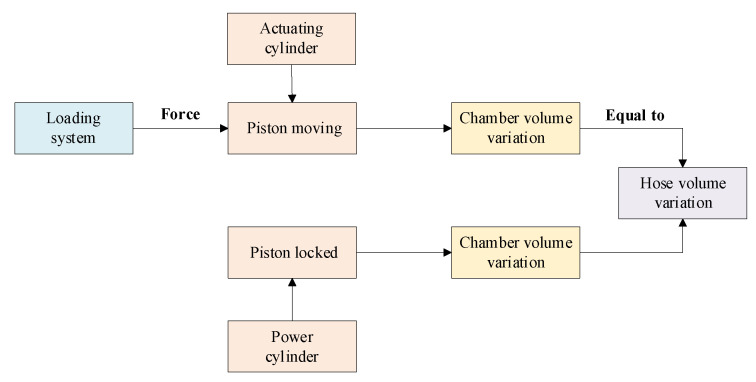
Data acquisition block diagram for hose chamber characteristics.

Since the piston of the actuating cylinder is mechanically fixed, the volume of its rodless chamber changes while the corresponding volume of the power cylinder remains unchanged (Figure 6). Consequently, it can be assumed that both the rodless chamber and the tested hose have the same volumetric variation. It can be written as:

(24) $${\dot{V}}_{h1}=A\cdot {\dot{X}}_{o}$$

Equation (25) defines the bulk elastic modulus of the oil. The mathematical model of the hose can be established after acquiring the pressure and volume change in the tested hose.

(25) $$\beta_{e}=-V\frac{\Delta P}{\Delta V}$$

**Figure 7:**
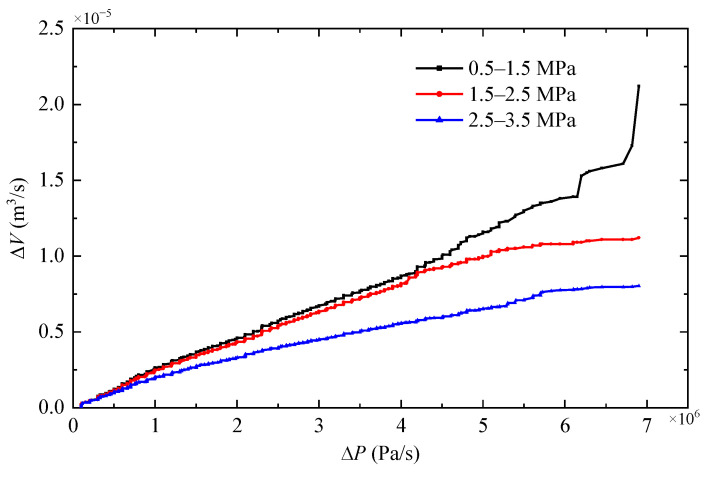
Curves of Δ*V* versus Δ*P*.

When the pressure rises at the same rate, both the elastic coefficient and the volume change rate of the hose decrease. In order to avoid the adverse effect of the decrease in elastic coefficient on the model accuracy, the operation pressure is divided to three sections including 0.5–1.5 MPa, 1.5–2.5 MPa, and 2.5–3.5 MPa. Each section accommodates 840 sets of data as illustrated in Figure 7.

**Figure 8:**
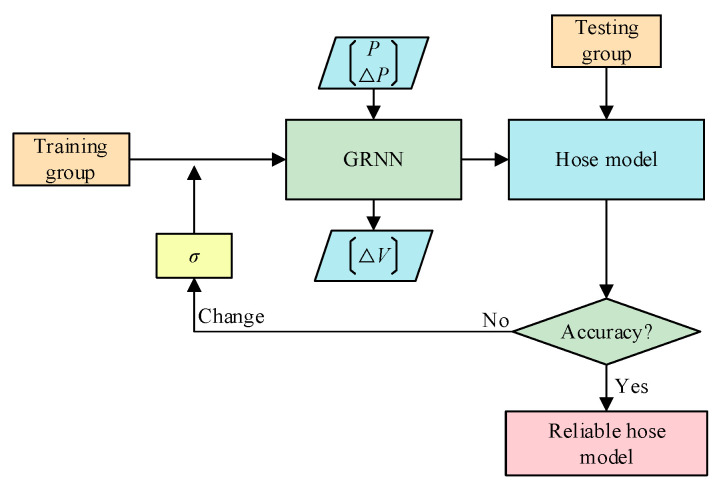
GRNN training process.

GRNN-based hose modeling process is shown in Figure 8. The input to the network consists of the pressure and the rate of its change. The output is the rate of the hose volume. The neural network is trained through the training group and the testing group. If the test accuracy of the network does not meet the requirement, the training is repeated by an updated σ until the expected hose model is obtained. The prediction accuracy can be calculated by:

(26) $$V_{Acc}=\sum_{i=1}^{n}\bar{\left(1-\frac{\left|V_{Ti}-V_{Pi}\right|}{V_{Ti}}\right)}\cdot 100\%$$

where *V_Acc_* is the prediction accuracy of the volume; *V_Ti_* is the ith test sample value; and *V_Pi_* is the ith predictive value.

**Figure 9:**
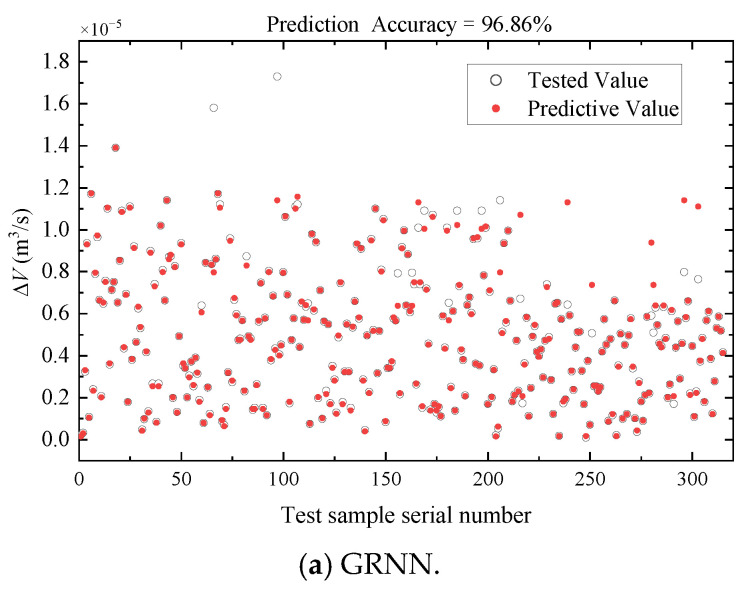

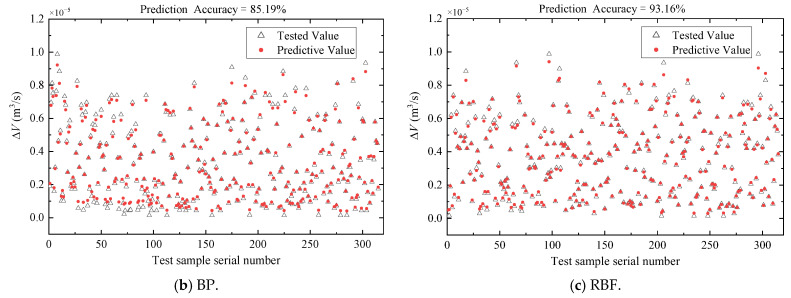
Prediction accuracy for the training group.

**Figure 10:**
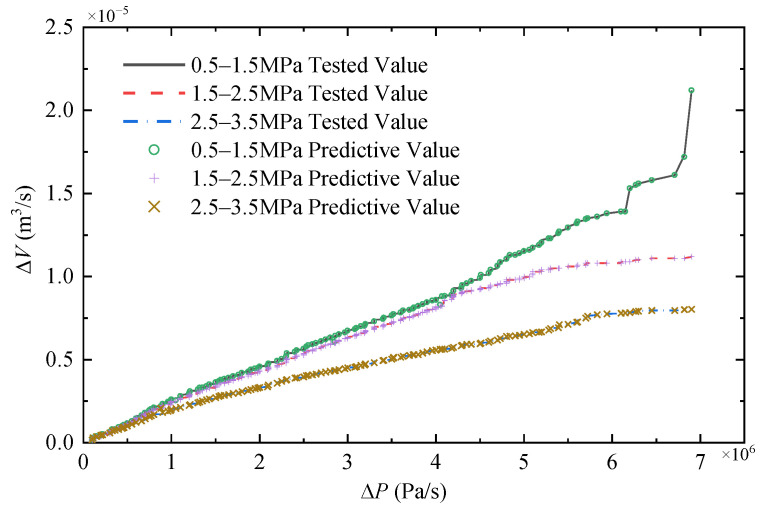
Prediction results for three pressure sections.

**Figure 11:**
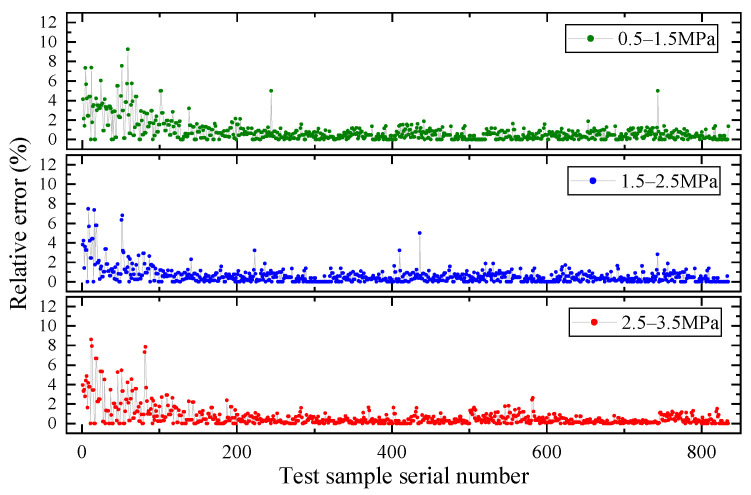
Relative errors for three pressure sections.

In this study, we compared the performance of three neural network models, GRNN, RBF, and BP, to predict the deformation of flexible hoses. The results are shown in Figure 9. It can be seen that GRNN performed the best in terms of prediction accuracy, followed by RBF. During the training process, we observed that BP and RBF models had disadvantages in parameter adjustment and training time. Considering the overall performance, including accuracy and training process, we selected GRNN as the preferred model for modeling the hoses. Finally, let *σ* = 0.1. In addition, all the data from the three sections are inputted into the GRNN-based hose model and the predicted change rate of the volume is shown in Figure 10. The relative errors of the predicted results are shown in Figure 11, with average errors of no more than 5% for all three sections.

It can be seen from Figure 10 and Figure 11 that the GRNN-based hose model can well-identify the volume change in the hose, which can contribute to a more accurate model of the controlled object for sequential studies on controllers’ design.

## 4. Design of Generalized MPC

### 4.1. Control Structure

**Figure 12:**
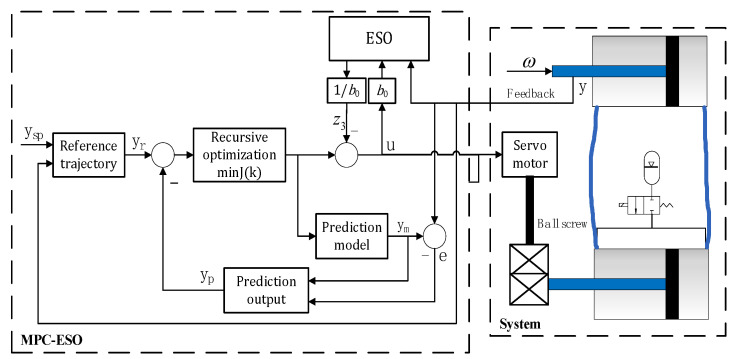
Position control system based on MPC-ESO.

Figure 12 shows the control structure of a double-cylinder hydraulic actuating system. The MPC control algorithm is used to calculate the optimal piston control sequence of the power cylinder in the future. Then, the minimum difference between the piston position of the actuator and the desired position is considered as the performance index subject to the mathematical model and constraints of the double-cylinder hydraulic actuating system.

The first element of the control sequence is calculated as the actual servo motor control variable. Then, the prediction horizon moves one step forward to repeat this process at the next time step. The ESO estimates the external disturbance on the system.

In Figure 12, *y*_sp_ represents the input of the system, namely, the expected output position of the actuating cylinder piston; *y*_r_ represents the tracking reference; *y*_m_ is the output of the actual controlled double-cylinder actuating system using GRNN to build the hose model; *y*_p_ is the predicted output, and the augmented minimal state-space equation of the double-cylinder actuation system using the lumped parameter method to build the hose model is used as the prediction model; *ω* is the external disturbance, namely, the load applied on the actuating cylinder piston; and *u* is the output of the controller to the motor. The actual position of the actuating cylinder is fed back to the controller by the LVDT.

### 4.2. Design of Augmented State-Space MPC

The continuous system is first discretized [27]. Then, the state variables and control quantities are selected according to Equation (12), and the static error is eliminated in increments, as follows:

(27) $$\left\{\begin{array}{l} \Delta X(k+1)=A_{c}\Delta X(k)+B_{c}\Delta U(k)\\ Y\left(k\right)=C_{c}\Delta X(k)+Y\left(k-1\right) \end{array}\right.$$

where *k* is the sampling time and *A_c_*, *B_c_*, and *C_c_* are the coefficient matrices of the discrete system with the sampling period *T_s_*.

Adding the integral in the state-space model, the augmented matrix can be obtained as:

(28) $$A=[\begin{array}{cc} A_{c} & O_{d}\\ C_{c}A_{c} & 1 \end{array}],B=[\begin{array}{c} B_{c}\\ C_{c}B_{c} \end{array}],C=[\begin{array}{cc} O_{d} & 1 \end{array}]$$

where *A*, *B,* and *C* are the corresponding augmented matrices of *A_c_*, *B_c_*, and *C_c_*; *O_d_* = [0,0,…,0]_1 × *n*_.

At the sampling moment *k_i_*, the control trajectory can be written as Δ*u*(*k_i_*), Δ*u*(*k_i_* + 1), ..., Δ*u*(*k_i_* + *N_c_* − 1), where *N_c_* is the dimension of the control trajectory vector. With the sampling starting point *k_i_* and the initial state variable *x*(*k_i_*), the subsequent state variables are written as:

(29) $$\begin{array}{l} x\left(k_{i}+1|k_{i}\right)=Ax\left(k_{i}\right)+B\Delta u\left(k_{i}\right)\\ x\left(k_{i}+2|k_{i}\right)=A^{2}x\left(k_{i}\right)+AB\Delta u\left(k_{i}\right)+B\Delta u\left(k_{i}+1\right)\\ x\left(k_{i}+m|k_{i}\right)=A^{m}x\left(k_{i}\right)+A^{m-1}B\Delta u\left(k_{i}\right)+\cdots +A^{m-N_{c}}B\Delta u\left(k_{i}+N_{c}-1\right)\\ x\left(k_{i}+N_{p}|k_{i}\right)=A^{N_{p}}x\left(k_{i}\right)+A^{N_{p}-1}B\Delta u\left(k_{i}\right)+\cdots +A^{N_{p}-N_{c}}B\Delta u\left(k_{i}+N_{c}-1\right) \end{array}$$

where *x*(*k_i_* + *m*|*k_i_*) is the predicted state variable at *k_i_* + *m* moment and *N_p_* is the dimension of the predicted trajectory vector. The output variables are written as:

(30) $$\begin{array}{l} y\left(k_{i}+1|k_{i}\right)=CAx\left(k_{i}\right)+CB\Delta u\left(k_{i}\right)\\ y\left(k_{i}+2|k_{i}\right)=CA^{2}x\left(k_{i}\right)+CAB\Delta u\left(k_{i}\right)+CB\Delta u\left(k_{i}+1\right)\\ y\left(k_{i}+N_{p}|k_{i}\right)=CA^{N_{p}}x\left(k_{i}\right)+CA^{N_{p}-1}B\Delta u\left(k_{i}\right)+\cdots +CA^{N_{p}-N_{c}}B\Delta u\left(k_{i}+N_{c}-1\right) \end{array}$$

According to the equations above, the output vector can be written as:

(31) $$Y=\left[y\left(k_{i}+1|k_{i}\right)\right.y\left(k_{i}+2|k_{i}\right)y\left(k_{i}+3|k_{i}\right)\cdots {\left.y\left(k_{i}+N_{p}|k_{i}\right)\right]}^{T}$$

The control variables can be expressed as:

(32) $$\Delta U=\left[\Delta u\left(k_{i}\right)\right.\Delta u\left(k_{i}+1\right)\Delta u\left(k_{i}+2\right)\cdots {\left.\Delta u\left(k_{i}+N_{c}-1\right)\right]}^{T}$$

Combining Equation (32) with Equation (31), we have

(33) $$Y=F\cdot x\left(k_{i}\right)+\Phi \Delta U$$

where, $F={\left[CA,\;CA^{2},\;\cdots ,\;CA^{N_{p}}\right]}^{T}$,

$$\Phi =\left[\begin{array}{cccc} CB & 0 & \cdots & 0\\ CAB & CB & \cdots & 0\\ \vdots & \vdots & & \vdots \\ CA^{N_{p}-1}B & CA^{N_{p}-2}B & \cdots & CA^{N_{p}-N_{c}}B \end{array}\right]$$

At the sampling time of *k_i_*, for a given *r*(*k_i_*), the goal of the prediction control is to make the actuating cylinder piston position as close as possible to the given signal in a prediction horizon. This requires finding an optimal control vector Δ*U* that minimizes the error function between the given signal and the predicted output.

Establishing the optimization objective function is defined by:

(34) $$\begin{array}{l} \min J={\left(R_{s}-Y\right)}^{T}(R_{s}-Y)+\Delta U^{T}\bar{R}\Delta U\\ s.t.\;\;U^{\min}\leq C_{1}u(k_{i}-1)+C_{2}\Delta U\leq U^{\max}\\ \;\;Y^{\min}Fx(k_{i})+\Phi \Delta U\leq Y^{\max} \end{array}$$

(35) $$R_{s}={\left[\begin{array}{cccc} 1 & 1, & \cdots & ,1 \end{array}\right]}_{N_{p}\times 1}^{T}\times r(k)$$

where *R_s_* is the target value matrix and $\bar{R}$ is the compensation matrix.

The objective function and constraint conditions are arranged by the standard form of the quadratic programming method as:

(36) $$\begin{array}{l} \min\Delta U^{T}(\Phi^{T}\Phi +\bar{R})\Delta U-2\Delta U^{T}\Phi^{T}(R_{s}-Fx(k_{i}))\\ s.t.\;\;\;\;\;\;\;\;\;\;M\Delta U\leq \eta \end{array}$$

where *M* is the constraint matrix of the system; *N_c_* is the control horizon; and *η* is the constraint vector. They can be written as:

$$M=\left[\begin{array}{c} -C_{2}\\ C_{2}\\ -\Phi \\ \Phi \end{array}\right],\;\eta =\left[\begin{array}{c} -U^{\min}+C_{1}u(k_{i}-1)\\ U^{\max}+C_{1}u(k_{i}-1)\\ -Y^{\min}+Fxk_{i}\\ Y^{\max}-Fxk_{i} \end{array}\right],C_{1}={\left[\begin{array}{c} 1\\ 1\\ \vdots \\ 1 \end{array}\right]}_{N_{c}\times 1}$$

The optimization problem can be formulated as a Quadratic Program (QP) with linear inequality constraint. The optimal input sequence at *k_i_* moment can be solved according to the quadratic optimization algorithm.

(37) $$\Delta U={\left(\Phi^{T}\Phi +\bar{R}\right)}^{-1}\Phi^{T}\left(R_{s}-Fx\left(k_{i}\right)\right)$$

Let ${\bar{R}}_{s}={\left[\begin{array}{cccc} 1 & 1, & \cdots & ,1 \end{array}\right]}_{N_{p}\times 1}^{T}$, Equation (37) can be written as:

(38) $$\Delta U={\left(\Phi^{T}\Phi +\bar{R}\right)}^{-1}\Phi^{T}\left({\bar{R}}_{s}r\left(k_{i}\right)-Fx\left(k_{i}\right)\right)$$

According to the rolling time-domain control principle, at *k_i_* moment, only the first element of Δ*U* acts as the controlled increment:

(39) $$\begin{array}{l} \Delta u\left(k_{i}\right)={\left[\begin{array}{cccc} 1 & 0 & \cdots & 0 \end{array}\right]}_{1\times N_{c}}{\left(\Phi^{T}\Phi +\bar{R}\right)}^{-1}\left(\Phi^{T}{\bar{R}}_{s}r\left(k_{i}\right)-\Phi^{T}Fx\left(k_{i}\right)\right)\\ \Delta u^{*}\left(k_{i}\right)=K_{y}r\left(k_{i}\right)-K_{mpc}x\left(k_{i}\right) \end{array}$$

where $\Delta u^{*}(k_{i})$ stands for the system input at *k_i_* moment; *K_y_* is the first element of ${\left(\Phi^{T}\Phi +\bar{R}\right)}^{-1}\Phi^{T}{\bar{R}}_{s}$; and *K_mpc_* is the state feedback gain vector and the first line of ${\left(\Phi^{T}\Phi +\bar{R}\right)}^{-1}\Phi^{T}F$.

Using Equation (39) combined with the extended state equation, the system prediction controller can be obtained as:

(40) $$x\left(k+1\right)=(A-BK_{mpc})x(k)+BK_{y}r(k)$$

Due to the special structure of the matrices *C* and *A*, the last column of the matrix *F* is [1 1 … 1]^T^ equal to ${\bar{R}}_{s}$.Therefore, *K_y_* is the same as the last element of *K_mpc_*. Considering the state variable $x(k)=[\Delta x_{m}{\left(k\right)}^{T}y{\left(k\right)}^{T}]$, the state feedback gain vector is *K_mpc_* = [*K_x_ K_y_*], where *K_x_* and *K_y_* are the feedback gain vectors of Δ*x_m_*(*k*)^T^ and *y*(*k*), respectively.

**Figure 13:**
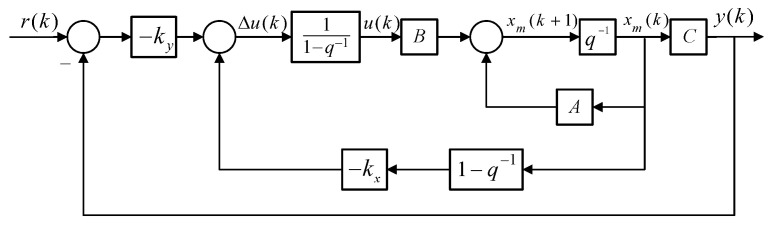
Block diagram of model predictive control.

The block diagram of the model predictive control is shown in Figure 13. Here, *q*^−1^ is the time backward shift operator.

### 4.3. Design of ESO

The extended state observer predicts and compensates the total disturbance of the system. A nonlinear second-order ESO with single input and single output for the double-cylinder actuation system is written as:

(41) $$\left\{\begin{array}{l} {\dot{X}}_{o}=\gamma \\ \dot{\gamma }=f\left(\gamma ,a,b\right)+b_{0}u+\omega \left(t\right)\\ u={\dot{X}}_{i}\\ y={\dot{X}}_{o} \end{array}\right.$$

where *ω*(t) is the external disturbance and *f*(*γ*,*a*,*b*) is the internal disturbance. The piston speed of the power cylinder ${\dot{X}}_{i}$ and the displacement piston of the actuating cylinder *X_o_* are the input and output of the actuation system, respectively.

The nonlinear state observer is:

(42) $$\left\{\begin{array}{l} e=z_{1}-y\\ {\dot{z}}_{1}=z_{2}-\beta_{1}e\\ {\dot{z}}_{2}=z_{3}-\beta_{2}fal\left(e,\alpha_{1},\delta \right)+b_{0}u\\ {\dot{z}}_{3}=-\beta_{3}fal\left(e,\alpha_{2},\delta \right) \end{array}\right.$$

where *z*_1_ and *z*_2_ represent the observed piston displacement of the actuating cylinder and the observed piston speed of the power cylinder, respectively; *z*_3_ is the estimated disturbance; *u* is the control input; *b*_0_ is the compensation factor; *α*_1_ and *α*_2_ are the tuned parameters in (0,1); *β*_1_, *β*_2_, and *β*_3_ are the observer gains; and *fal* is the nonlinear function of the ESO as in Ref. [28].

(43) $$fal\left(e,\alpha ,\delta \right)=\left\{\begin{array}{l} e/\delta^{\left(1-\alpha \right)},\;\;\;\;\;\;\left|e\right|\leq \delta \\ {\left|e\right|}^{\alpha }\cdot sign(e),\;\left|e\right|>\delta \end{array}\right.$$

Considering the stability of the ESO, the following assumptions are made. First, the internal and external disturbance function *f*(*γ*,*a*,*b*), *ω*(t) are continuously differentiable with respect to their variables and

(44) $$\left|u\right|+\left|f\left(\gamma ,a,b\right)\right|+\left|\omega \left(t\right)\right|+\left|\frac{\partial f}{\partial X_{o}}\right|\leq c_{0}+\sum_{j=1}^{n}c_{j}{\left|X_{o}\right|}^{k}$$

where *c_j_*, *j* = 0,1, …, *n* denote the positive constants and *k* is the positive integer.

Second, *ω*(t) and the solution *X_o_* of (32) meet:

(45) $$\left|\omega \left(t\right)\right|+\left|X_{o}\left(t\right)\right|\leq B_{0}$$

where *B*_0_ is the positive constant, *i* = 1, 2,…,n, and *t* ≥ 0.

Third, the following constants exist: *λ_i_* (*i* = 1, 2, 3, 4), *α*, *β,* and the positive definition, the continuous differentiable functions *V*, *W*: ${\mathbb{R}}^{n+1}\to \mathbb{R}$ in order that:

(46) $$\left\{\begin{array}{l} \lambda_{1}{\left\|y\right\|}^{2}\leq V\left(y\right)\leq \lambda_{2}{\left\|y\right\|}^{2}\\ \lambda_{3}{\left\|y\right\|}^{2}\leq W\left(y\right)\leq \lambda_{4}{\left\|y\right\|}^{2}\\ \sum_{i=1}^{n}\frac{\partial y}{\partial y_{i}}\left(y_{i+1}-g_{i}\left(y_{1}\right)\right)-\frac{\partial y}{\partial y_{n+1}}g_{n+1}\left(y_{1}\right)\leq -W\left(y\right)\\ \left|\frac{\partial y}{\partial y_{n+1}}\right|\leq \xi \left\|y\right\| \end{array}\right.$$

where *y* = (*y*_1_, *y*_2_,…, *y_n_*_+1_), $\left\|\cdot \right\|$ denotes the Euclid norm of ${\mathbb{R}}^{n+1}$.

If all the above assumptions are tenable, for every positive constant *a*, uniformly in $t\in \left[a,\infty \right)$.

(47) $$\begin{array}{l} \underset{\beta \to 0}{\lim}\left|X_{o}\left(t\right)-z_{i}\left(t\right)\right|=0\\ \bar{\underset{t\to \infty }{\lim}}\left|X_{o}\left(t\right)-z_{i}\left(t\right)\right|\leq O\left(\beta^{n+2-i}\right) \end{array}$$

Accordingly, the stability of ESO is confirmed. The proof of the theorem is available in Ref. [28]. The main idea of this algorithm is to transform the error of the object system using the ESO into an asymptotical stable system with a small disturbance, in order to eliminate the effect of the total disturbance error by the high gain of the observer.

## 5. Experimental System

### Double-Cylinder Experimental Bench

**Figure 14:**
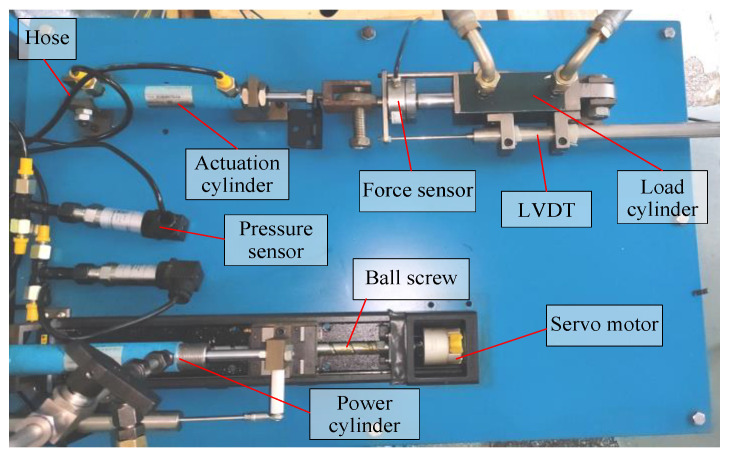
Double-cylinder experimental bench.

To obtain the data for the GRNN and verify the effects of the MPC strategy, experimental studies are conducted on the double-cylinder experimental bench. The arrangement of the system is given as shown in Figure 14. It consists of a servo motor, a ball screw, two hydraulic cylinders, two pressure sensors, a LVDT, two force sensors, and any other software or hardware modules that are required.

**Figure 15:**
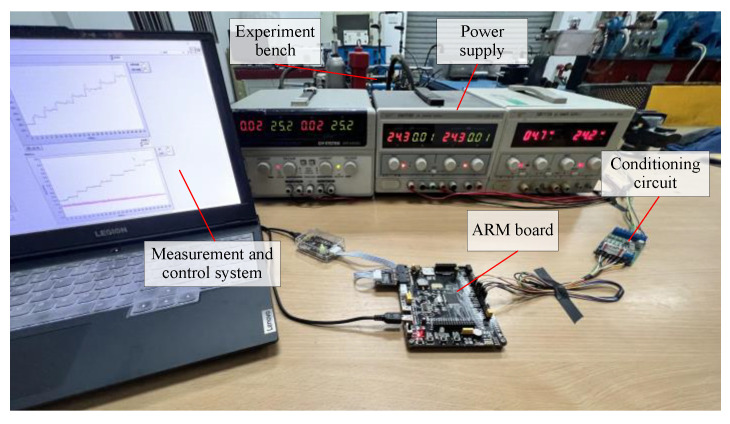
Measurement and control system.

The measurement and control system are shown in Figure 15. It includes a host computer and C code-based ARM STM32F417VET board. In the ARM board, A/D converter reads the sensor output through the signal conditioning circuit. Compared with the command from the control program through the USART serial port, the designed control strategy calculates the PWM output to the motor encoder, and then drives the motor.

## 6. Results

### 6.1. Model Validation

**Figure 16:**
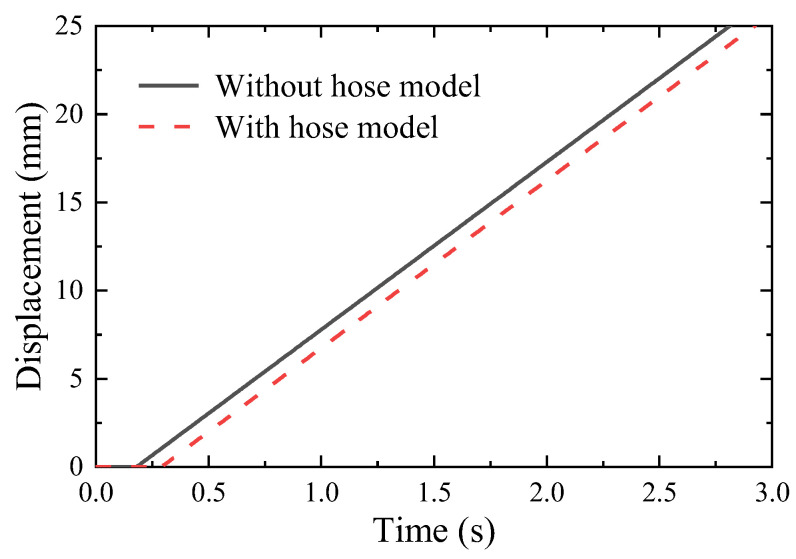
Displacement of the actuating cylinder.

In order to qualitatively evaluate the reliability of the hose model, Figure 16 compares the open-loop simulation results of the system with and without the hose model. It implies that during the system startup, the position of the actuating cylinder with the hose has a long response lag, since it takes the hose more time to build the initial pressure in the system.

**Figure 17:**
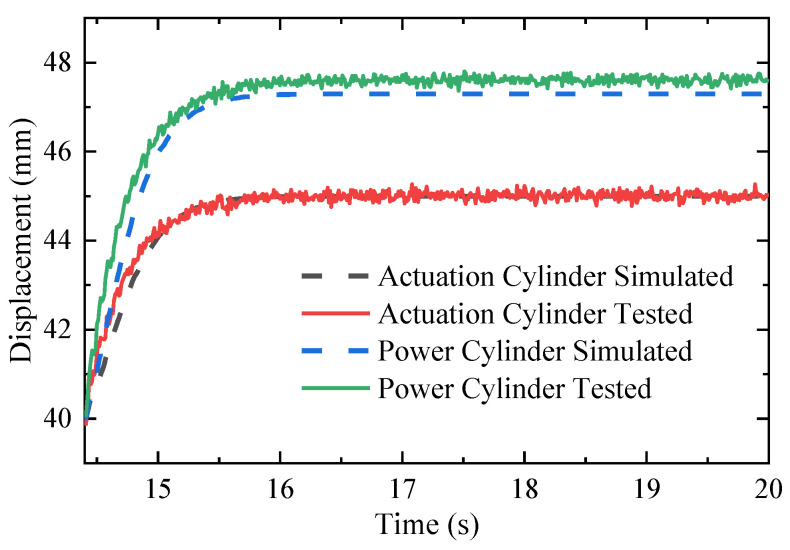
Displacement of two cylinders.

In order to confirm the reliability of the mathematical model for the whole actuation system, a preliminary experiment is implemented on the system as shown in Figure 14. Figure 17 illustrates the position response of the two cylinders to 45 mm command. The experimental displacement of the power cylinder is 2.3 mm longer than the actuating cylinder, and the simulated displacement of the power cylinder is less than the tested result. The inconsistency of the two cylinders can be attributed to the combined effects of hose expansion, oil compression, and leakage in the pressurized chamber.

### 6.2. Load-Free Displacement Response

To verify the dynamic performance effectiveness of the proposed position control strategy, some parameters of the system and the controllers are listed in Table 1 and Table 2, respectively.

**Figure 18:**
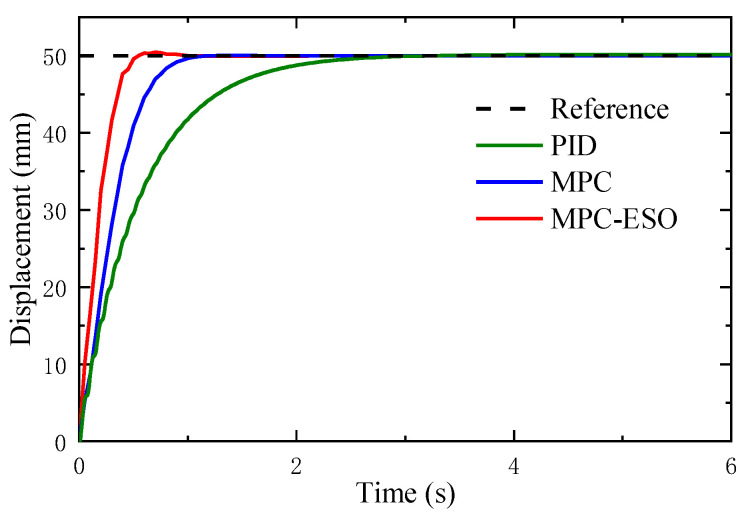
Responses to step command.

The effectiveness of the proposed strategy is evaluated by comparison among PID, traditional MPC, and MPC-ESO based on the augmented state-space prediction model. Figure 18 depicts the responses of the system with three different controllers to a step command. Similar to the response of a 50 mm step command, the system using MPC-ESO reaches a stable state in a shorter time. To provide a comprehensive comparison of the performance of MPC-ESO, MPC, and PID controllers in step response, Table 3 presents the quantitative performance data for these three controllers, including rise time, steady-state error, and square error. The data in the table clearly demonstrate the superior performance of the MPC-ESO controller in terms of shorter rise time and minimized steady-state error compared to the MPC and PID controllers.

**Figure 19:**
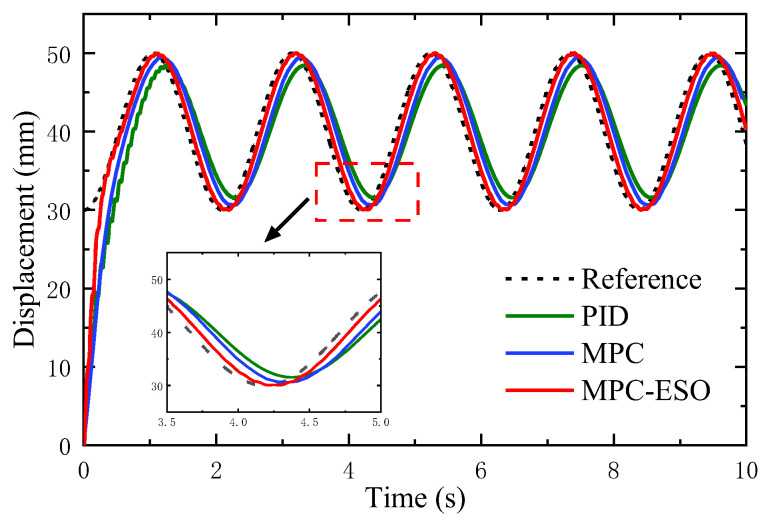
Responses to sinusoidal command.

In order to further study the effects of the MPC-ESO, sinusoidal tracking performance of the system using the strategy is simulated and the results are shown in Figure 19. Compared to the other two controllers, the system using the MPC-ESO can track the command with fast response.

The demonstration implies that the double-cylinder actuation system using the MPC-ESO strategy has great potential in response to speed and tracking precision improvement.

### 6.3. Displacement Response with Load

**Figure 20:**
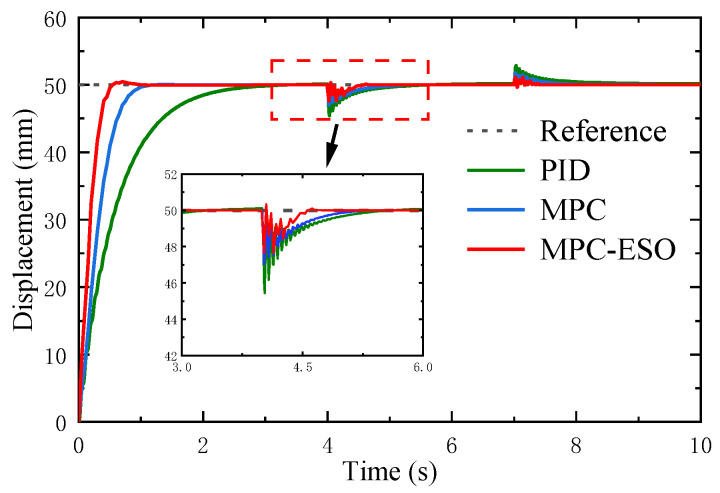
Responses to step load disturbance.

In this section, we present the simulation results of the controller’s anti-interference ability under different conditions. The performance of the three controllers under step load is shown in Figure 20. For a 50 mm command and a stepping load disturbance (0–800 N at 4 s and 800–400 N at 7 s), the actuation system using the MPC-ESO controller can realize the smallest position error. To present a comprehensive comparison of the disturbance rejection performance of MPC-ESO, MPC, and PID controllers under step load, Table 4 provides quantitative performance data for these three controllers, including overshoot, settling time, and Root Mean Square Error (RMSE). The data in the table clearly demonstrate the superior disturbance rejection capability of the MPC-ESO controller compared to the MPC and PID controllers. It exhibits significantly reduced overshoot, faster settling time, and lower RMSE, indicating its effectiveness in mitigating the impact of step load disturbances on the control system.

**Figure 21:**
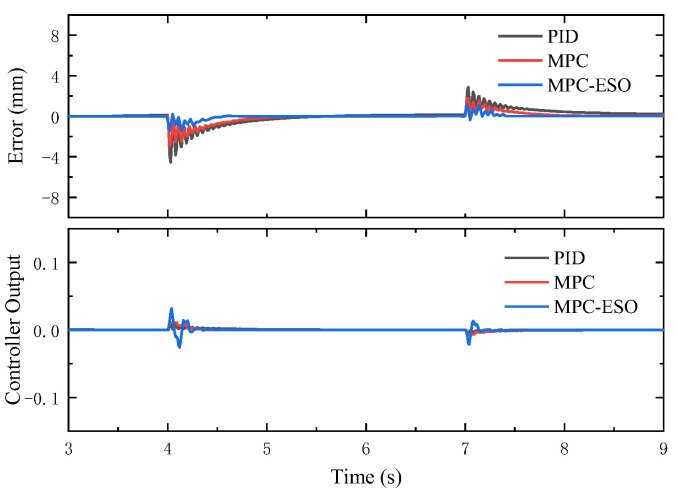
Error and controller output.

**Figure 22:**
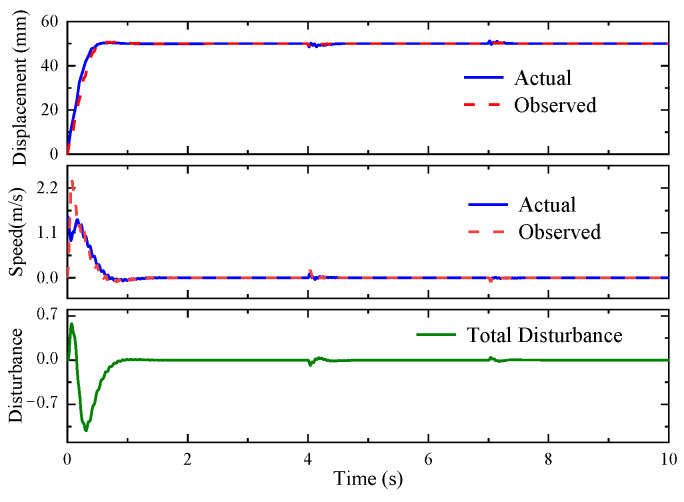
Observations by ESO.

To some extent, this is due to the fact that when the step load interferes with the system output, the MPC-ESO controller can produce faster and higher-amplitude control signal (as shown in Figure 21) to make the system response more quickly and reduce the disturbance impact on the system, as well. Combined with the MPC, the ESO obtains the observed value of the state variables and the disturbances. Figure 22 indicates that the observed results were consistent with the actual values.

**Figure 23:**
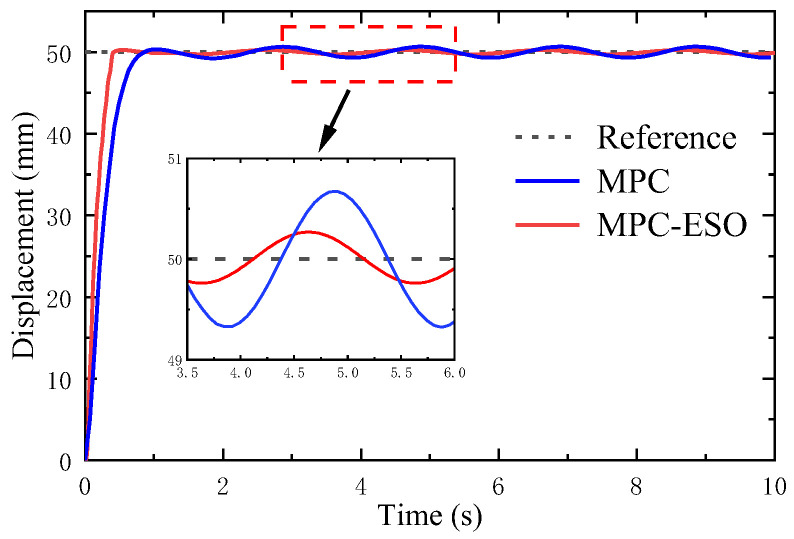
Response to a sinusoidal load.

**Figure 24:**
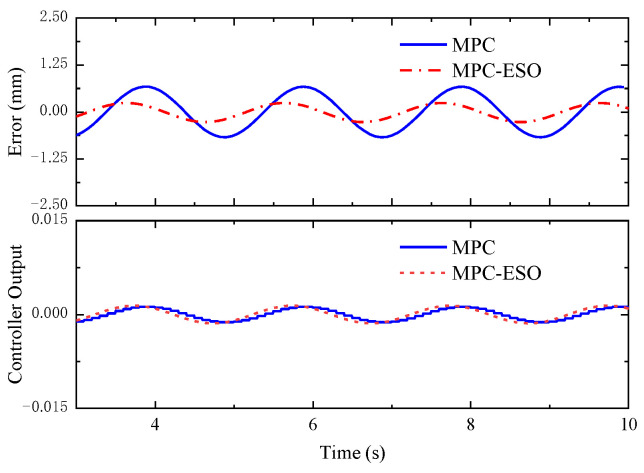
Error and controller output.

**Figure 25:**
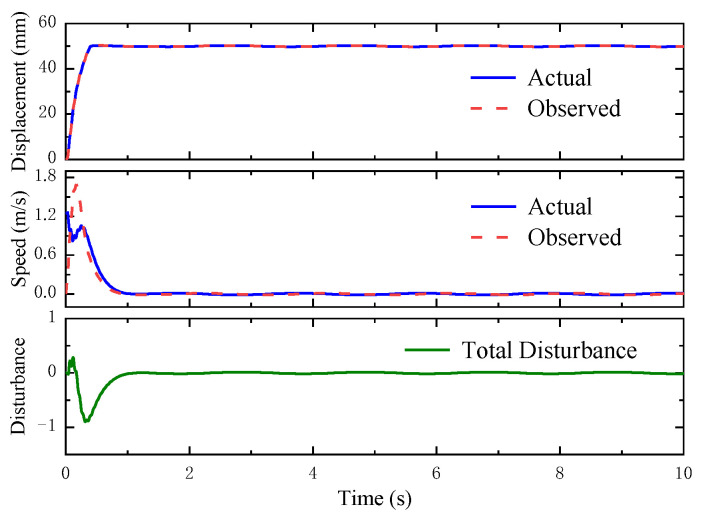
Observations by ESO.

Figure 23 depicts the step response to a sinusoidal load disturbance (*F_t_* = 200sin(πt) + 400 N). The MPC-ESO brings about a smaller steady-state error of the actuation system than the simple MPC, which confirms its better robustness. The displacement error and output by the two controllers is illustrated in Figure 24. The output of the MPC lags behind the MPC-ESO by 0.3 s. Figure 25 shows the observed speed, displacement, and total disturbance of the actuating cylinder via the ESO. These observed results are basically qualified and can lay a foundation of high-performance control of analogous uncertain systems.

### 6.4. Displacement Control Experiment

**Figure 26:**
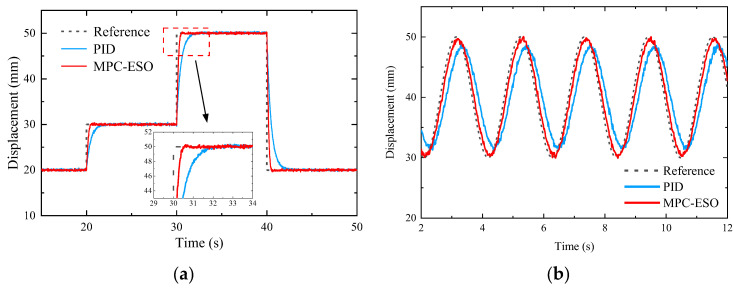
Position tracking. (**a**) Step response. (**b**) Sinusoidal response.

In this section, the proposed method is further verified in the double-cylinder actuator system. The parameters of the MPC-ESO controller are *N_p_*_2_ = 8, *N_c_*_2_ = 3, *β*_1_ = 25, *β*_2_ = 120, and *β*_3_ = 35, while *P* = 2.8 and *I* = 0.06 are for the PID controller. The position response performance of the proposed MPC-ESO is verified in Figure 26. Figure 26a shows the response to a step command. Compared to PID, MPC-ESO can well-track the expected position. The settling time is nearly 1 s and the steady-state error is less than 0.2 mm. The sinusoidal tracking result is shown in Figure 26b. With the MPC-ESO strategy, the actuating cylinder tracks the command with the minimum error of 0.8 mm. In general, the MPC-ESO controller is effective in achieving the high-precision position tracking for the small-scale actuation system.

**Figure 27:**
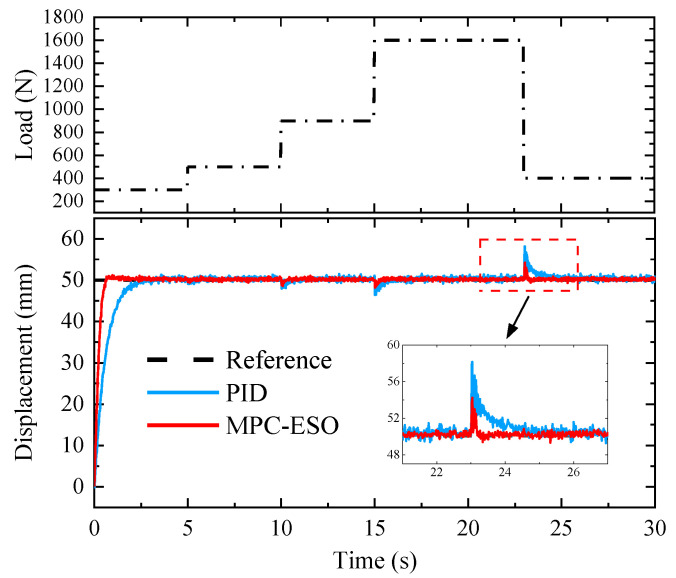
Step response under different loads.

The anti-disturbance capacity of the MPC-ESO control is experimentally verified. Figure 27 shows the position response with a step load exerted on the actuating cylinder. It can be observed that the settling time of the position controlled by the MPC-ESO is significantly less than that of PID under each step disturbance. For a disturbance force step from 1600 N to 400 N, the displacement oscillation meets the expected level with an amplitude of no more than 7%.

**Figure 28:**
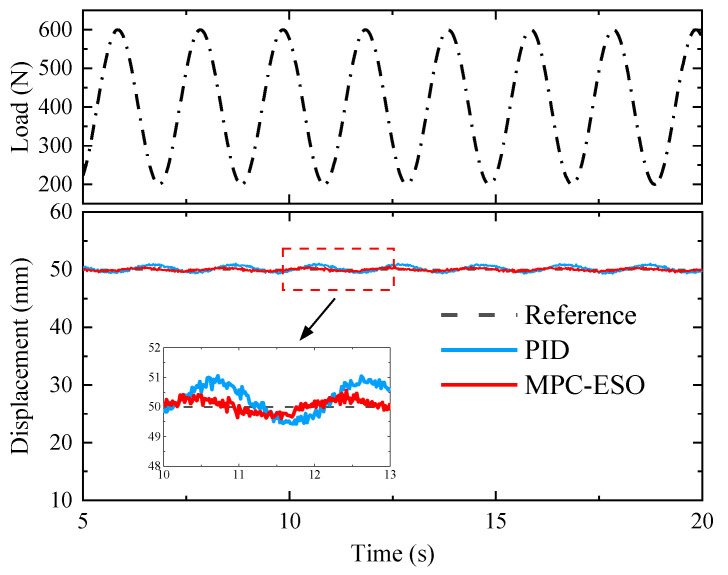
Step response under sinusoidal loads.

Further verification of the MPC-ESO strategy is conducted by applying sinusoidal force disturbance under a step command. Figure 28 shows the position performance of the controller under sinusoidal load. It can be seen that the system adopting the MPC-ESO controller has higher accuracy under the sinusoidal disturbance. The position error of the actuation cylinder does not exceed 3% of the expected output from the command. The experimental results demonstrate that the MPC-ESO strategy is effective in achieving high-precision and high-dynamic position tracking for the small-scale actuation system.

The results of the position control system based on the MPC-ESO strategy in the experiment meet the expectation of simulation.

## 7. Conclusions

This study proposes the MPC-ESO strategy for displacement control of a miniature double-cylinder actuation architecture. GRNN-based hose representation is assessed to improve the system model. Considering the uncertainty and no model aspects, the hose experiment has been carried out to obtain the pressure-volume increment data. Based on this, GRNN is adopted to better represent the volumetric variation behavior of the hose filled with high-pressure oil. Considering the new hose model, the whole actuation system model is built and its reliability has been confirmed. Then, MPC-ESO strategy is designed to strengthen the anti-disturbance capability. Simulations on the system suffering from typical external disturbances have been accomplished to verify the effect of the proposed hybrid control strategy.

According to the results, the conclusions are drawn as follows:

(1) GRNN’s nonlinear mapping by using experimental data achieves accurate identification of volumetric variation of the hose (average errors do not exceed 5%) and improves the precision for the whole actuation system model.
(2) The proposed MPC-ESO hybrid control strategy effectively overcomes the impact of the model error, parameter, or environment uncertainties on the displacement control performance of the miniature double-cylinder actuation system. Additionally, it can shorten the response duration and tracking lag, and decrease the output oscillation of the system under load disturbances.

Simulations and experiments demonstrate that the double-cylinder actuation system adopting the MPC-ESO strategy allows for higher control precision and better anti-disturbance capability.

## Figures and Tables

**Table 1:** System parameters.

Parameter	Value	Parameter	Value
m	50 kg	r	0.003 m
A*_P_*_1_	3.77 × 10^−4^ m^2^	l	1 m
A*_P_*_2_	4.91 × 10^−4^ m^2^	σ	0.1
A*_A_*_1_	3.14 × 10^−4^ m^2^	k_2_	1 × 10^−6^
A*_A_*_2_	2.01 × 10^−4^ m^2^	μ*_visc_*	200 N·s/m

**Table 2:** Controller parameters.

Parameter	Value	Parameter	Value	Parameter	Value
P	2.7	N*_P_*_2_(MPC-ESO)	8	h	0.006
I	0.05	N*_c_*_2_(MPC-ESO)	3	h_0_	0.005
N*_p_*_1_(MPC)	6	β_1_	30	b	8
N*_c_*_1_(MPC)	2	β_2_	100	b_0_	0.02
T*_S_*	0.1 s	β_3_	40		

**Table 3:** Quantitative performance.

Step Response			
	MPC-ESO	MPC	PID
Rise time	0.75 s	1.2 s	3.1 s
Steady-state error	0 mm	0 mm	0.12 mm
Square error	0.0065 mm^2^	0.01 mm^2^	0.69 mm^2^

**Table 4:** Quantitative performance.

Step Load Response			
	MPC-ESO	MPC	PID
Overshoot	2.82%	5.9%	9.12%
Settling Time	0.65 s	1.2 s	2.1 s
RMSM	0.81 mm	1.64 mm	2.2 mm

## Data Availability

The data that support the findings of this study are available on request from the corresponding author.
